# Metformin-induced caveolin-1 expression promotes T-DM1 drug efficacy in breast cancer cells

**DOI:** 10.1038/s41598-018-22250-8

**Published:** 2018-03-02

**Authors:** Yuan-Chiang Chung, Ching-Ming Chang, Wan-Chen Wei, Ting-Wei Chang, King-Jen Chang, Wei-Ting Chao

**Affiliations:** 1 Department of Surgery, Cheng-Ching General Hospital, Chung-kang Branch, Taichung, Taiwan; 2 Division of Hematology and Oncology, Cheng-Ching General Hospital, Chung-kang Branch, Taichung, Taiwan; 3 Department of Life Science, Tunghai University, Taichung, Taiwan; 4 Department of Surgery, Taiwan Adventist Hospital, Taipei, Taiwan

## Abstract

Trastuzumab emtansine (T-DM1) is an antibody drug conjugate (ADC) that was recently approved for the treatment of HER-2-positive metastatic breast cancer. The drug sensitivity of ADCs depends mainly on the internalization efficiency of the drug. Caveolin-1 was shown to promote T-DM1 internalization and enhance drug sensitivity. Whether caveolin-1 can be overexpressed to improve T-DM1 efficacy is interesting and has the potential for clinical application. In this study, diabetes drug metformin was investigated in terms of induction of caveolin-1 expression for increased efficacy of subsequent T-DM1 application. BT-474 cells were pretreated with metformin, followed by combined therapy with metformin and T-DM1. The T-DM1 internalization and drug efficacy were determined, and the protein expressions for signal transduction were also monitored. Caveolin-1 shRNA was applied to suppress endogenous caveolin-1 expression, and the ability of metformin to promote T-DM1 efficacy was investigated. Result showed that in BT-474 cells pretreated with metformin, cellular caveolin-1 overexpression was induced, which then promoted drug efficacy by enhancing T-DM1 internalization. As cellular caveolin-1 was suppressed by shRNA, the effect of metformin-enhanced T-DM1 cytotoxicity was decreased. This study demonstrated that metformin can be applied prior to T-DM1 treatment to improve the clinical efficacy of T-DM1 by enhancing caveolin-1-mediated endocytosis.

## Introduction

Trastuzumab emtansine (trastuzumab-DM1; T-DM1) was recently developed as a new-generation target drug for breast cancer. T-DM1 is a trastuzumab (Herceptin)-based antibody drug conjugate (ADC) that is conjugated to emtansine, which can prevent microtubule assembly. The trastuzumab in T-DM1 can bind to HER-2 receptors, followed by internalization of T-DM1 into cells; emtansine is then released, resulting in cell toxicity^1,2^. T-DM1 significantly prolonged the progression-free and overall survival in metastatic breast cancer patients, with less toxicity, in phase III of the EMILIA trial^3^.

The drug efficacy of T-DM1 is dependent on cell endocytosis^4–7^. Recent studies demonstrated that caveolin-1, a 21KD membrane protein that plays a role in endocytosis and vesicle trafficking, is co-localized with trastuzumab to promote T-DM1 internalization and enhance drug efficacy^8,9^. The expression level of caveolin-1 is varied in patients, and is not correlated with HER-2 expression^9^. Therefore, T-DM1 may not work well in HER-2-positive patients with low caveolin-1 expression.

T-DM1 was reported to exert a significant benefit in terms of survival in patients with HER-2-positive advanced breast cancer previously treated with trastuzumab and a taxane in a second-line study^3^. However, in the recent MARIANNE trial^10^, the progression-free survival (PFS) under T-DM1 treatment was found to be non-inferior, but not superior, to trastuzumab plus a taxane as the first-line treatment for local advanced or metastatic breast cancer. Although T-DM1 treatment did not result in significant improvement of PFS, subgroup analyses showed a numerical trend of an increasing beneficial effect of T-DM1 in patients who had received HER-2-directed therapy or taxanes during early treatment^10^. Similar results were also obtained in the TH3RESA trials^11^. However, the mechanism of T-DM1 in improving the clinical outcome in patients previously treated with trastuzumab or taxanes remains elusive. Furthermore, whether expression of caveolin-1 could be induced by treatment with trastuzumab or taxanes in breast cancer cells to obtain a greater beneficial effect of T-DM1 treatment was also assessed in the current study.

In previous studies, the first-line diabetes drug metformin, which can inhibit mitochondria ATP production through up-regulation of AMPK, was demonstrated to induce caveolin-1 expression in lung and breast cancer cells^12,13^, and caveolin-1 is required for AMPK activity when metformin is applied. Therefore, whether metformin-mediated caveolin-1 overexpression can improve T-DM1 efficacy in breast cancer cells was examined in this study. Our study assessed the caveolin-1 expression upon treatment with metformin, and the drug efficacy of T-DM1 after caveolin-1 induction was also determined.

## Results

### Pretreatment with trastuzumab improves T-DM1 efficacy

**Figure 1:**
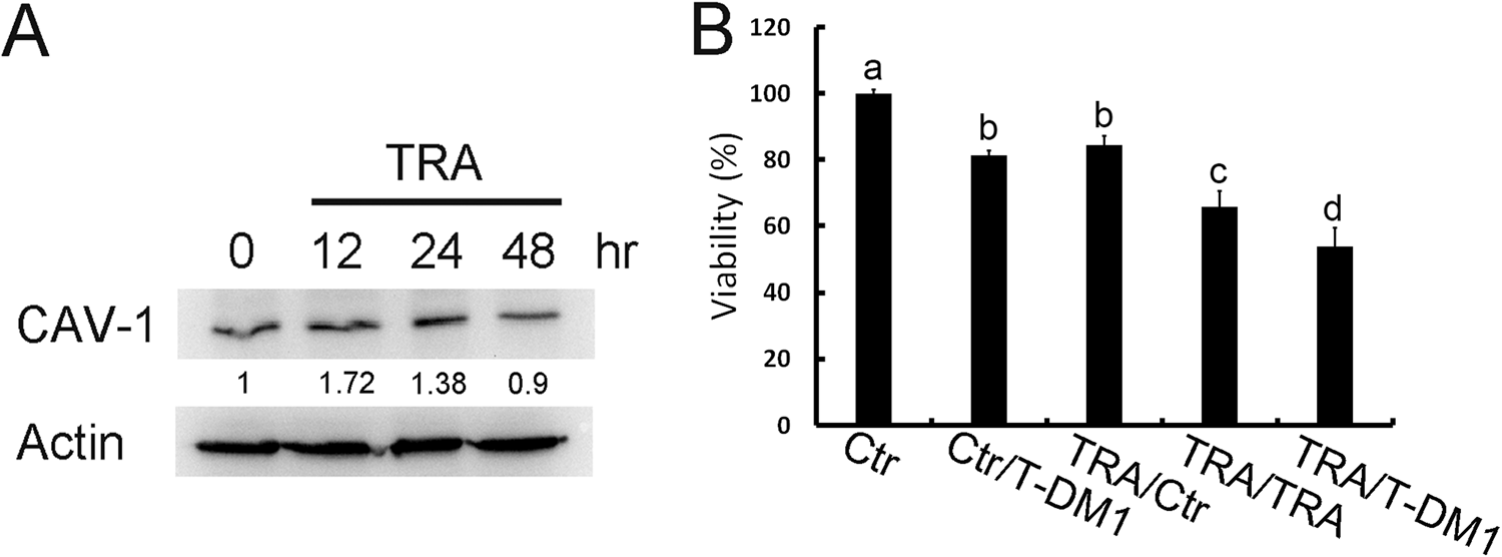
Pretreatment with trastuzumab in HER-2-positive cells up-regulated caveolin-1 expression and improved T-DM1 drug efficacy. (**A**) Treatment of BT-474 cells with trastuzumab (10 μg/ml) induced caveolin-1 overexpression at 12 and 24 hours. (**B**) BT-474 cells pretreated with trastuzumab (TRA) for 24 hours were then treated with trastuzumab or T-DM1 for an additional 72 hours, and the cell viability was significantly decreased. Ctr: control group without treatment; TRA: trastuzumab. Statistical values with different letters were significantly different (*P* < 0.01). At least three independent experiments were performed for all quantifications. The western blots were derived under the same experimental conditions from the same cell lysates; the original full-length Western blot images are showed in Supplementary Figure S1.

BT-474 cells are HER-2-positive breast cancer cells. The results of our previous study demonstrated that caveolin-1 expression is necessary for T-DM1 uptake in BT-474 cells^9^. In this study, BT-474 cells were treated with trastuzumab, which up-regulated the overexpression of caveolin-1 peaking at around 12–24 hours (Fig. 1A). To investigate whether induced caveolin-1 can promote T-DM1 efficacy, the pretreatment strategy was applied in this study. When T-DM1 was applied to trastuzumab-pretreated BT-474 cells, the cytotoxic effect was improved significantly as compared with trastuzumab or the T-DM1 treatment (Fig. 1B). These results suggested that trastuzumab-induced caveolin-1 expression may be critical prior to T-DM1 treatment.

### Metformin induces caveolin-1 expression and promotes T-DM1 efficacy

**Figure 2:**
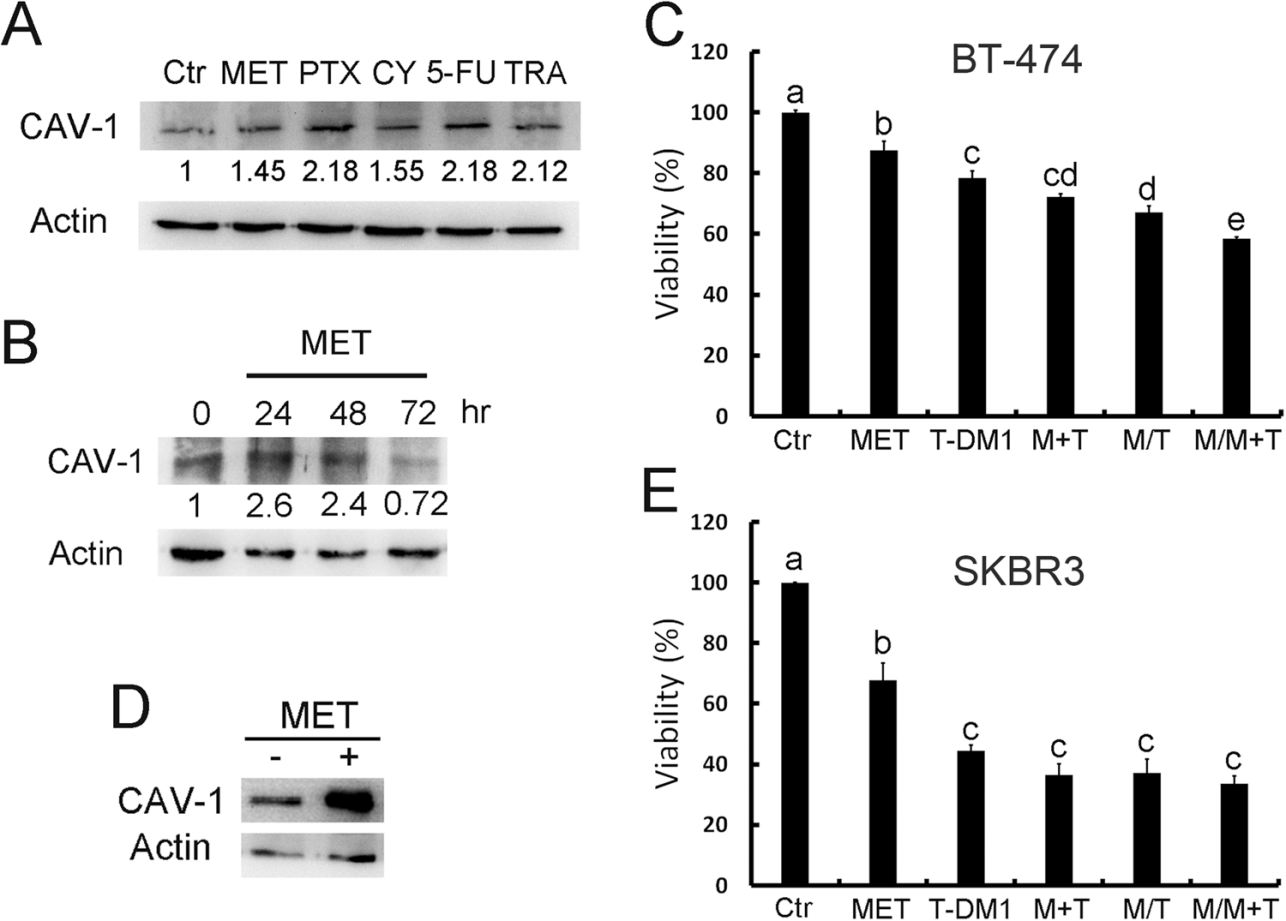
Metformin induced caveolin-1 expression and promoted T-DM1 efficacy. Western blot analysis demonstrated that when BT-474 cells were treated with metformin and other chemotherapy drugs, caveolin-1 expression was induced (**A**); this started to decrease at 72 hours when treated with metformin (**B**). (**C**) In BT-474 cells pretreated with metformin for 24 hours and then treated with a combination of T-DM1 and metformin, the cell viability was significantly decreased. (**D**) SKBR3 cells treated with metformin for 24 hours, caveolin-1 expression was induced. (**E**) SKBR3 treated with metformin combined with T-DM1 or pretreated with metformin and followed by T-DM1 treatment with or without metformin combination showed decreased cell survival compared to metformin or T-DM1 treated group. MET/M: metformin; PTX: paclitaxel; CY: cyclophosphamide; 5-FU: 5-fluorouracil; TRA: trastuzumab; T: T-DM1. Statistical values with different letters were significantly different (P < 0.01). At least three independent experiments were performed for all quantifications. The western blots were derived under the same experimental conditions from the same cell lysates of each treatment group in (**A,B** and **D**); the original full-length western blot images are showed in Supplementary Figure S2.

**Figure 3:**
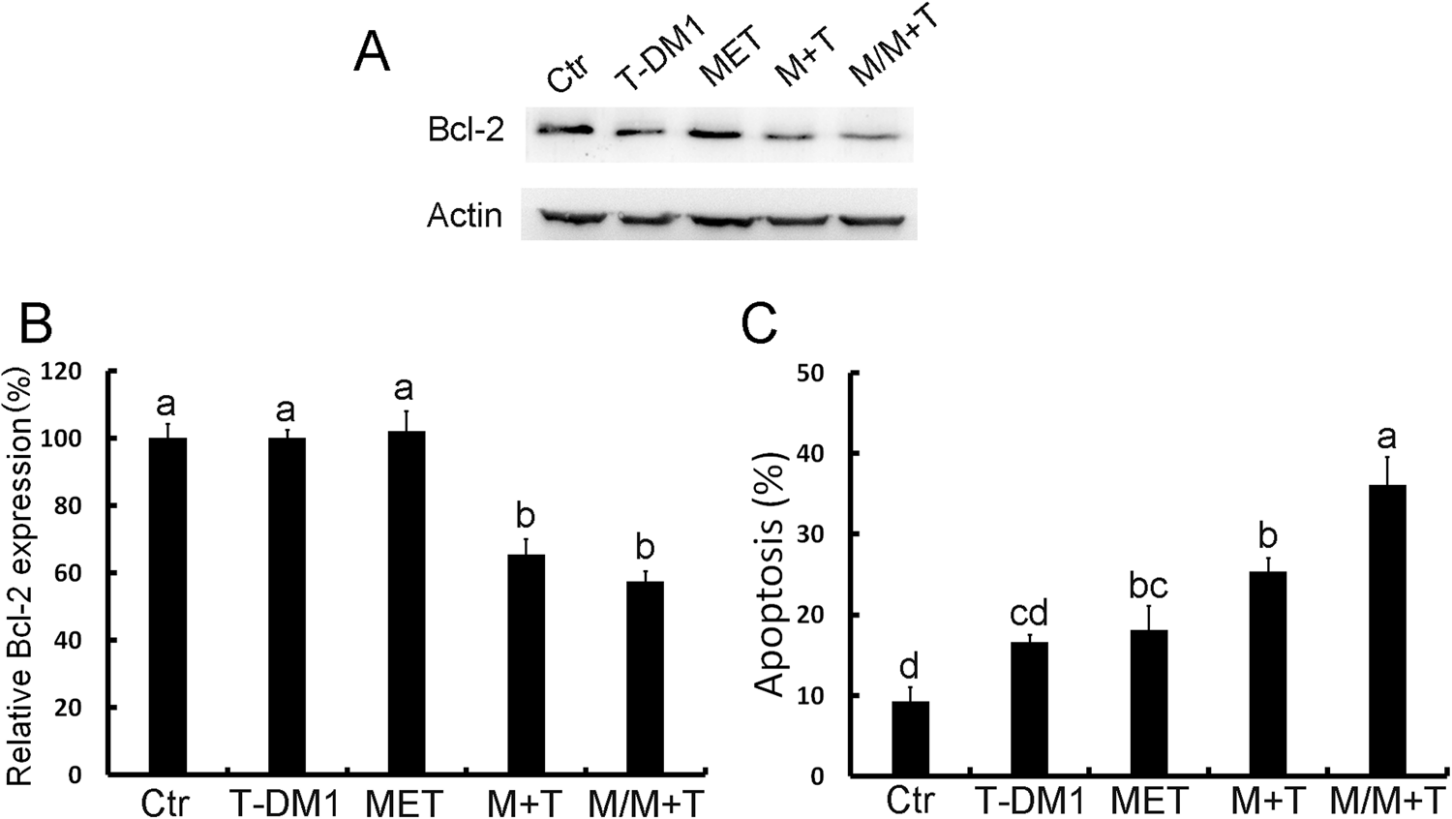
Pre-treatment with metformin promoted T-DM1 efficacy through apoptosis. (**A**,**B**) Western blot analysis showed that when BT-474 cells were pretreated with metformin and then treated with a combination of T-DM1 and metformin, the Bcl-2 level was significantly decreased. (**C**) Cell staining with annexin V/PI for a cell apoptosis assay also demonstrated that pretreatment with metformin followed by treatment with T-DM1/metformin induced cell apoptosis dramatically. MET/M: metformin; T: T-DM1. Statistical values with different letters were significantly different (*P* < 0.01). At least three independent experiments were performed for all quantifications. The western blots were derived under the same experimental conditions from the same cell lysates; the original full-length western blot images are showed in Supplementary Figure S3.

As the induction of caveolin-1 expression can improve T-DM1 efficacy, we then applied different drugs and examined their effects on caveolin-1 expression. According to our data, the first-line diabetes drug metformin elicited induction of caveolin-1 in BT-474 cells, peaking at around 24 to 48 hours (Fig. 2A,B). The pretreatment strategy was applied to examine whether metformin induced caveolin-1 can promote T-DM1 efficacy. Because metformin is the diabetes medicine for regular use, the continue dose was applied with T-DM1 treatment to evaluate the benefit of drug combination after metformin pre-treatment. In the result, when cells were pretreated with metformin and then treated with a combination of metformin and T-DM1, the cell viability was decreased obviously as compared with solitary treatment with metformin or T-DM and a control group (Fig. 2C). The other HER-2 positive cell SKBR3 was also examined, the caveolin-1 expression was also induced after metformin treatment (Fig. 2D), when metformin and T-DM1 were applied; drug efficacy was also improved but did not show statistic difference compared to T-DM1 treatment (Fig. 2E). Since the SKBR3 has relative high caveolin-1 expression being sensitive to T-DM1 treatment^9^, the effect of metformin was not so prominent in T-DM1 treatment. The apoptosis-associated protein Bcl-2 was also monitored by western blot analysis, the results of which showed that in BT-474 cells pretreated with metformin followed by combination treatment with T-DM1 and metformin, the level of pro-survival protein Bcl2 was significantly decreased (Fig. 3A,B). The status of apoptosis was also determined by annexin V/PI staining, which showed increasing apoptotic activity in BT-474 cells pretreated with metformin and then treated with combined metformin and T-DM1 (Fig. 3C). These results further indicated that metformin treatment up-regulated caveolin-1 expression and improved T-DM1 efficacy through an apoptotic mechanism.

### Metformin pretreatment enhances T-DM1 internalization

**Figure 4:**
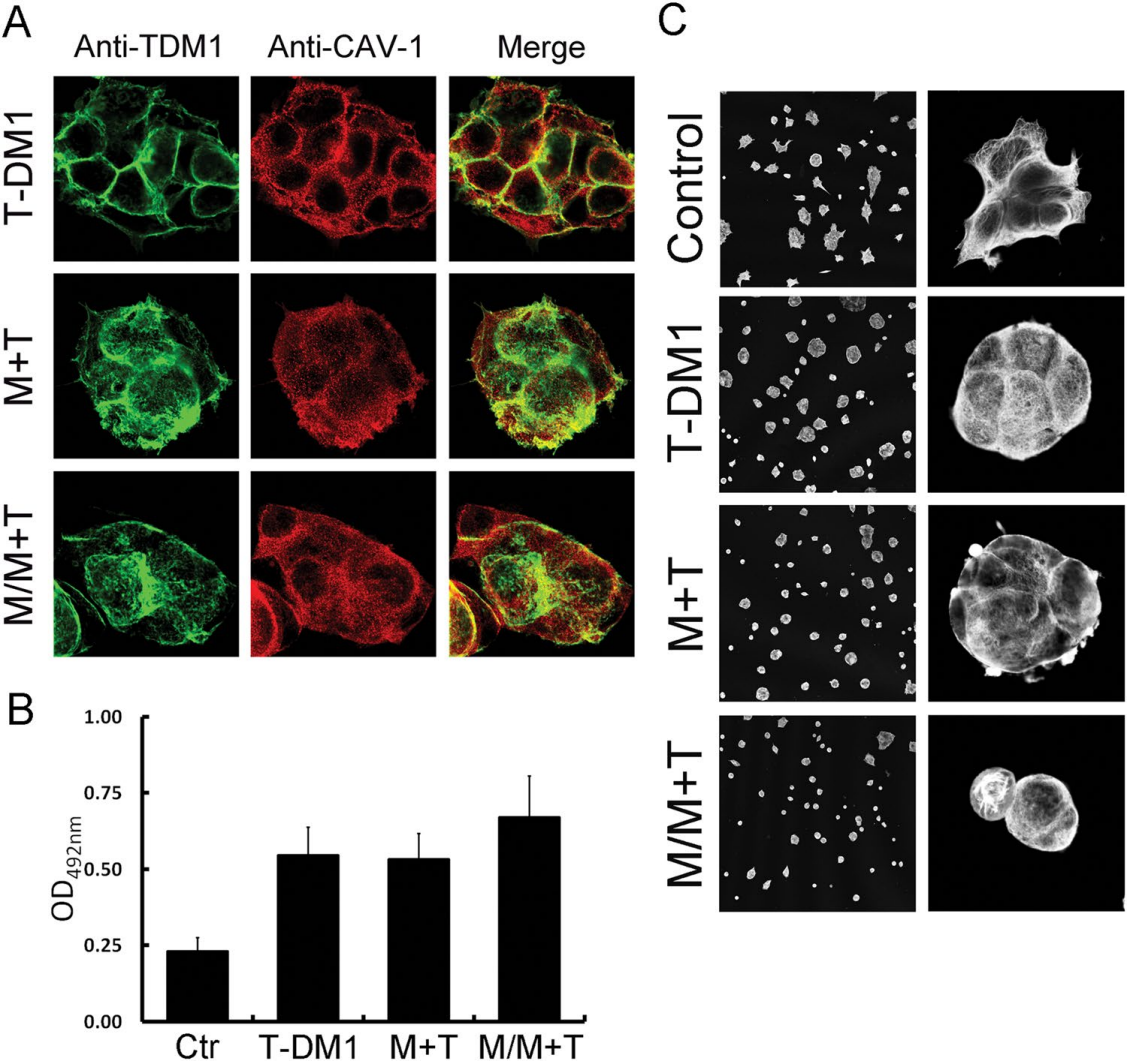
Metformin treatment changed the caveolin-1 distribution and promoted T-DM1 internalization. (**A**) Immuno-confocal microscopy showed that when BT-474 cells were treated with metformin/T-DM1 or pretreated with metformin and followed by metformin/T-DM1 treatment, caveolin-1 (red) and T-DM1 (green) were co-localized from the cell membrane to the cytoplasm. (**B**) A membrane biotinylation assay showed that T-DM1 internalization in BT-474 cells was increased after pretreatment with metformin. (**C**) Immuno-confocal microscopy showed that the tubulin structure collapsed after T-DM1 treatment, and the shape of the cells became more spherical; when cells were co-treated or pre-treated with metformin, the tubulin structure was disrupted, and cells were detached and the morphology turned round. Left panel, low magnification; right panel, high magnification. Ctr: control; M: metformin; T: T-DM1.

To further clarify whether metformin-induced caveolin-1 expression enhanced T-DM1 internalization, the localization of T-DM1 was tracked by immunofluorescence microscopy. Confocal imaging showed that BT-474 cell pretreatment with metformin resulted in caveolin-1 redistribution; localization in the cytosol increased and T-DM1 internalization was enhanced (Fig. 4A). Quantification of the colorimetric results showed that pretreatment with metformin resulted in increased T-DM1 internalization in BT-474 cells (Fig. 4B). T-DM1 can induce cell toxicity through the activity of DM1, which conjugates with the trastuzumab antibody; DM1 is a tubulin inhibitor that prevents tubulin assembly, resulting in tubulin collapse. When BT-474 cells were pretreated with metformin followed by combined treatment with metformin and T-DM1, the intracellular tubulin structure was disrupted, resulting in detachment of the cell morphology (Fig. 4C). By visualization of T-DM1 internalization, metformin was found to induce caveolin-1 expression and T-DM1 internalization, and the release of DM1 was also confirmed by disruption of the tubulin structure.

### Combination of metformin and T-DM1 down-regulates MAPK and AKT activation through caveolin-1

**Figure 5:**
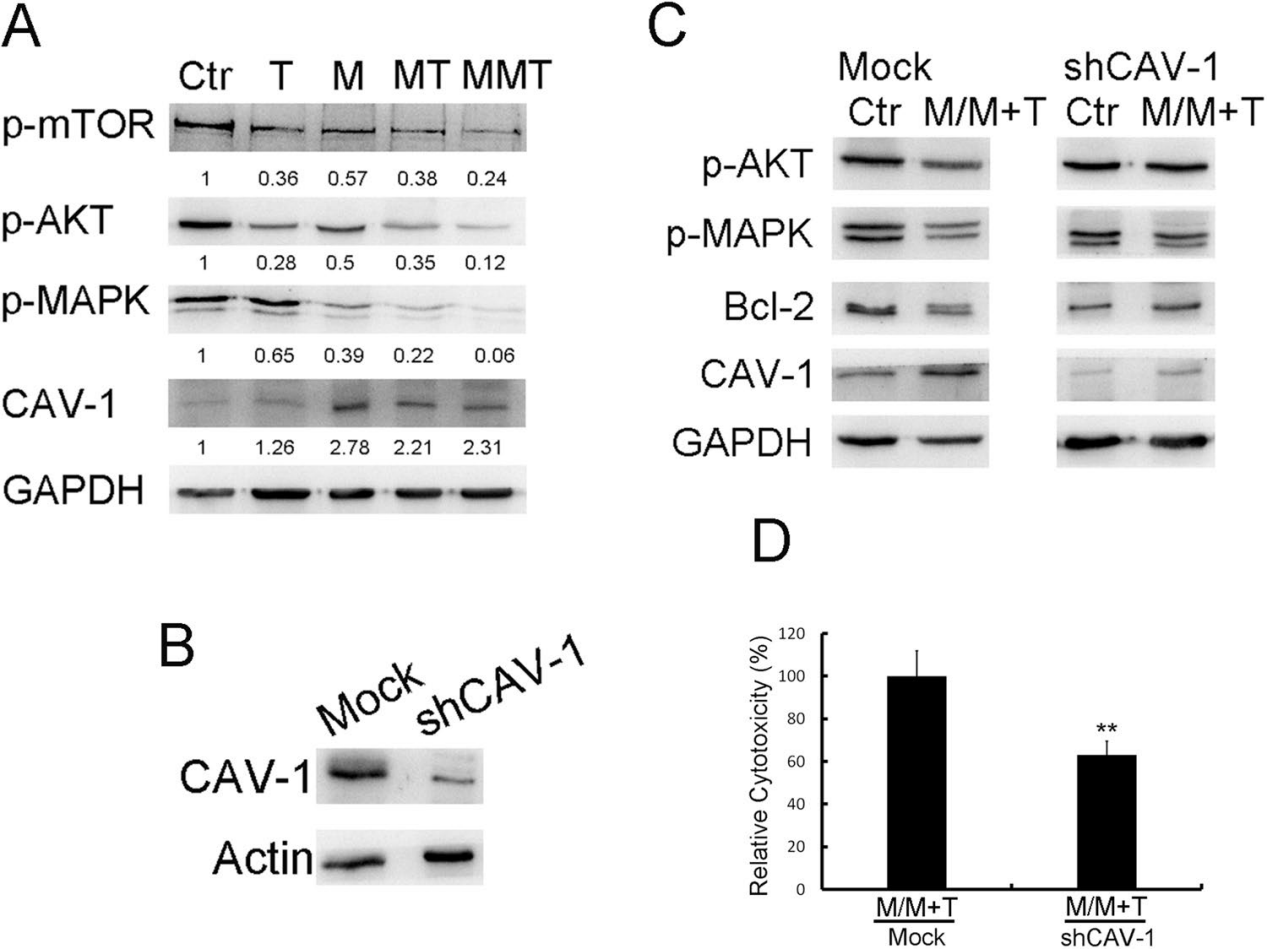
Cell signal mechanism of metformin pretreatment in terms of enhancement of T-DM1 efficacy. (**A**) The western blot results demonstrated that when BT-474 cells were pretreated with metformin and then treated with metformin in combination with T-DM1, the levels of phosphor-mTOR, AKT and MAPK were decreased. The original full-length western blot images are showed in Supplementary Figure S4. (**B**,**C**) Cells were pre-treated with caveolin-1 shRNA to create caveolin-1-deficient cells. Then, cells were treated with metformin followed by T-DM1 and metformin combined treatment. The data showed that in caveolin-1 knockdown cells, p-AKT and Bcl2 were not decreased, while p-MAPK was slightly affected. The original full-length western blot images are showed in Supplementary Figure S5. (**D**) Cells prepared as in (**C**) were subjected to MTT assay. In comparison with the mock group, which also underwent M/MT treatment, the cytotoxic effect was significantly reduced in caveolin-1 knockdown cells. shCAV-1: caveolin-1 shRNA; Ctr: control; M: metformin; T: T-DM1. Value = mean ± SD, *P < 0.05. The western blots were derived under the same experimental conditions from the same cell lysates of each treatment group in (**A**–**C**).

BT-474 cells were pretreated with metformin and then treated with T-DM1 or a combination of metformin and T-DM1, and cell lysates were collected and subjected to western blotting to detect mTOR, MAPK and AKT expressions. The results showed that metformin itself suppressed MAPK activity, and when metformin-pretreated cells were further treated with T-DM1 or combined metformin and T-DM1 treatment, mTOR, AKT and MAPK were decreased as compared with other single-treatment groups (Fig. 5A). To further identify whether metformin-induced caveolin-1 expression is necessary for T-DM1 efficacy, treatment was carried out on caveolin-1-knock-down BT-474 cells (Fig. 5B). The results of western blot analysis showed that when caveolin-1-deficient BT-474 cells were treated with metformin, followed by combined treatment with metformin and T-DM1, caveolin-1 expression was not induced, and the expressions of p-AKT and Bcl-2 were not suppressed after treatment (Fig. 5C), while p-MAPK was also slightly inhibited due to effect of metformin as mention in Fig. 5A. Similar molecular patterns were also observed in caveolin-1-deficient BT-474 cells that treated with metformin or trastuzumab (Supplementary Figure S6). Furthermore, when cells were pretreated with metformin followed by combined treatment with T-DM1 and metformin without caveolin-1, the cytotoxic effect was inhibited significantly (40%) (Fig. 5D). Internalization was also monitored by immunostaining; the confocal images showed the caveolin-1 knocked down BT-474 cells pretreated with metformin could inhibit the T-DM1 internalization (Supplementary Figure S7). From the molecular mechanism and the cellular toxicity results, expression of caveolin-1 was demonstrated to be critical in terms of the efficacy of metformin with T-DM1 treatment.

## Discussion

Breast cancer is a major type of cancer worldwide, and overexpression of human epidermal growth factor receptor 2 (HER-2), a marker for poor prognosis, is identified in 20–30% invasive breast cancers^14,15^. Trastuzumab (Herceptin), the humanized monoclonal antibody against HER-2 receptor, was approved by the Food and Drug Administration (FDA) in the USA for the treatment of patients with advanced breast cancers that express HER-2^16^. A combination of paclitaxel and trastuzumab was also demonstrated to be a good standard treatment for HER-2-positive breast cancer^17,18^.

As the MARIANNE trial demonstrated T-DM1 to be a non-inferior treatment resulting in a better quality of life as compared with trastuzumab plus a taxane, the National Comprehensive Cancer Network (NCCN) panel included T-DM1 as one of the first-line options for the treatment of patients with HER-2-positive metastatic breast cancer in 2016. However, pertuzumab, trastuzumab and a taxane remains the preferred front-line regimen for HER-2-positive metastatic disease. T-DM1 as a first-line therapy should be considered only in patients in which the preferred treatment is not suitable. Ongoing studies are being performed to evaluate antibody drug conjugates as an adjuvant therapy. However, identification of ways in which to increase the response rate of treatment with T-DM1 is still an important issue to be investigated prior to clinical trials, because in patients who achieve a T-DM1 treatment response, the duration of the response is longer than that seen in patients treated with trastuzumab plus a taxane^10^. Our previous study results demonstrated that caveolin-1 plays an important role in T-DM1 uptake; therefore, we hypothesized that a high expression of caveolin-1 may be an important factor in terms of the response to T-DM1 treatment^9^. Ligand such as antibody induced endocytosis and vesicle trafficking has been studied; antibody binds membrane receptor may trigger cellular vesicle function^19,20^. Other study and our previous study also demonstrated caveolin-1 is important and response for trastuzumab internalization^8,9^. In this study, we demonstrated that caveolin-1 could be induced in BT-474 cells by treatment with trastuzumab or a taxane. This result may explain why T-DM1 exerts a significant effect on survival in patients with HER-2-positive advanced breast cancer previously treated with trastuzumab and a taxane, as demonstrated in the EMILIA trial. On the contrary, T-DM1 treatment did not result in a superior PFS in comparison with the trastuzumab-containing regimen as a first-line treatment in the MARIANNE trial, possibly due to caveolin-1 expression not being induced sufficiently in treatment-naïve patients.

In current study, trastuzumab induced caveolin-1 expression and improved T-DM1 efficacy; however, using trastuzumab to induce caveolin-1 expression is not economic for clinical application, therefore, alternative ways in which to induce caveolin-1 expression and improve T-DM1 uptake are recommended in clinical T-DM1 therapy. Other than trastuzumab, paclitaxel has also been demonstrated to induce caveolin-1 expression^21^, as was also confirmed in our study, and caveolin-1 activation is necessary for paclitaxel efficacy. However, compare to the chemotherapeutic agents, other medicine “metformin” is safer and in our data also demonstrated the induction of caveolin-1 expression by metformin. Metformin is a low cost drug that used in the first line treatment of diabetes as oral form. Metformin increases the AMP/ATP ratio and promotes AMPK activation, and has been studied to assess its anti-tumor activity in breast cancer. The action of metformin on cancer cells depends on a direct effect mediated by AMP kinase and indirect action mediated by circulating insulin to decrease insulin receptor levels together with reduction in AKT and ERK 1/2 phosphorylation^22^. In the study of Salani and coworkers^13^, the sensitivity of cancer cells to metformin was found to be dependent on caveolin-1 expression, and metformin required caveolin-1 to induce AMPK phosphorylation. Therefore, in the current study, metformin was applied to increase caveolin-1 expression and thereafter improve T-DM1 sensitivity through apoptosis, and inhibition of MAPK pathway. These results are consistent with our previous study^9^.

Our results showed metformin could induce caveolin-1 expression. However, for keeping the high expression of caveolin-1 level during the time course of treatment of T-DM1, we continued to combine metformin usage with T-DM1 after pretreatment with metformin and gain the better efficacy. In clinical practice, the continuation of metformin might be also a modality of treatment in patients with diabetics to provide the benefit effect in T-DM1 therapy. Caveolin-1 expression was increased in the treatment with metformin; however, a phenomenon was demonstrated that caveolin-1 level decreased after the course of treatment of metformin combined with T-DM1. We suspected the cancer cells which possessed the high expression of caveolin-1 were sensitive to the T-DM1 cytotoxicity being killed during treatment, so the remaining cells showed less caveolin-1 expression. A contrary result was demonstrated in the study of Sung *et al*.^23^. They also approved T-DM1 internalization through caveolin-1 in gastric cancer cells. However, they showed high caveolin-1 expression is related to the acquired T-DM1 resistance. T-DM1 internalization is a major factor for drug efficacy, but the sensitivity to DM1 may vary in different cell types^1^.

Although metformin has a cytotoxic effect on cancerous cells, our data showed that knock-down of caveolin-1 expression suppressed the cytotoxic effect of treatment with T-DM1 combined with metformin by 40%. These results suggested that the role of metformin pretreatment in T-DM1 efficacy not only depends on the synergistic effect of metformin and T-DM1 in combination, but also on caveolin-1 expression induction for T-DM1 internalization. Combination of metformin and T-DM1 had inhibitory effects on p-MAPK and p-AKT expressions, and also increased apoptosis. However, with pretreatment with metformin to induce caveolin-1 expression followed by metformin and T-DM1 combined treatment, the drug efficacy in terms of molecular expression and cell toxicity was better than that observed with no pretreatment. Therefore, we suggest that pretreatment with metformin prior to metformin and T-DM1 combined treatment is recommended in clinical practice to gain a higher response rate to T-DM1; however, the definite benefits require further clinical investigation in the future for confirmation.

## Conclusions

The T-DM1 efficacy depends on the drug uptake ability by endocytosis. Previous reports have suggested negative correlations of caveolin-1 with human cancers^24,25^, and recent studies have proved that caveolin-1 inhibits breast cancer cell migration and metastasis^26–29^. Our study demonstrated for the first time that metformin can be used prior to T-DM1 treatment to induce caveolin-1 expression and improve drug uptake and increase the response to T-DM1. Diabetes is common in cancer patients; this study proves the benefits of metformin for the treatment of both diabetes and breast cancer patients treated with T-DM1. Currently, gene therapy is not a practical way in which to induce overexpression of caveolin-1 in breast cancer patients. This study provides an alternative method by which to induce caveolin-1 expression by metformin treatment. Taken together, our results suggested that enhancement of the efficacy of T-DM1 by pretreatment with metformin is a potential strategy for breast cancer treatment.

## Methods

### Cell line and drug treatments

BT-474 and SKBR3 breast cancer cell lines were purchased from the American Type Culture Collection (ATCC) (Manassas, VA, USA). The cell line was cultured with RPMI-1640 medium (Thermo Fisher Scientific, Waltham, MA, USA) containing 10% (v/v) fetal bovine serum (Thermo Fisher Scientific) and penicillin and streptomycin at 37 °C in a humidified 5% CO_2_ atmosphere.

Drug treatments: to examine cell viability, cells were plated in a 24-well plate at a density of 8 × 104 cells per well, and after 24 hours, 10 μg/ml trastuzumab (TRA; Roche Ltd., Basel, Switzerland) or 5 mM metformin (Sigma, St. Louis, MO, USA) was applied as a pretreatment for 24 hours. Cells were then treated with trastuzumab or T-DM1 (Kadcyla; Roche Ltd., Basel, Switzerland), or a combination of metformin and T-DM1 (1 ug/ml), depending on the experimental design^14^, for 72 hours. For western blot assay, 8 × 105 cells were seeded in 35-mm dishes and pretreated with 5 mM metformin, 0.1 μg/ml paclitaxel (PTX; Sinphar Ltd., I-Lan, Taiwan), 50 μM cyclophosphamide (CY; Sigma), or 1 μg/ml 5-fluorouracil (5-FU; MENARINI & C., Srl, Italy) for 24–72 hours. Metformin and T-DM1 treatments were applied for 48 hours after pretreatment.

For caveolin-1 knockdown, BT-474 cells were grown on 24-well plates in normal growth medium without antibiotics, and Lipofectamine 2000 transfection reagent (Invitrogen, ThermoFisher, Waltham, MA, USA) was used to transfect caveolin-1shRNA (RANi core; Academia Sinica, Taipei, Taiwan). Cells were analyzed 24 hr post-transfection, and the efficacy of transfection was confirmed by western blot analysis of cell lysates.

### Western blot

Cells were scraped with lysis buffer (1% NP-40, 50 mM Tris pH 7.4, 150 mM NaCl, 2 mM MgCl2, 1 mM EGTA, and protease and phosphatase inhibitors) on an ice tray, and cell lysates were subjected to western blot analysis. Protein samples were first separated by SDS–PAGE and then transferred to a PVDF membrane. Primary antibodies were applied to detect specific protein expressions, followed by incubation with appropriate HRP-conjugated secondary antibodies. Protein signals were developed using an enhanced chemiluminescence reagent (Millipore/Merck, Billerica, MA, USA) and detected by Multigel-21 digital system (Hung Chong, Taiwan) that revealed the relative intensity and compared to control group in the experiment. Western blots were carried out with caveolin-1 antibody (Santa Cruz, Dallas, TX, USA) and a marker for apoptosis was characterized using Bcl-2 antibody (Genetex, Irvine, CA, USA); markers for cell survival such as phosphor-mTOR, AKT, and MAPK antibodies (Cell Signaling Technology, Danvers, MA, USA) were also identified. Actin and GAPDH antibodies were used as loading control depending on the molecular weight of detecting proteins.

### Cell viability assay

Cell toxicity was determined by MTT assay and trypan blue staining in order to evaluate drug sensitivity. In the MTT assay, cells were seeded in a 24-well plate after drug treatment as described previously; 10 μl 5 mg/ml 3-(4,5-dimethylthiazol-2-yl)-2,5-diphenyltetrazoliumbromide (MTT; Sigma) solution (with 100 μl RPMI medium) were added to each well and incubated at 37 °C for 2 hours. The MTT solution was removed and the precipitated formazan crystals were dissolved in DMSO, following which the absorbance was recorded at 570 nm.

Cell viability was also determined with trypan blue staining. Briefly, 0.1 ml of 0.4% trypan blue and deionized water (1:1) were added to the cell suspension in order to estimate the number of dead cells. Cell viability was estimated using a hemocytometer. Dead cells were stained blue, while live cells remained unstained.

### Confocal microscopy for the detection of antibody internalization

Cells with or without caveolin-1 knockdown grown on glass coverslips were incubated with 5 mM metformin for 24 hours, then continued to be treated with 1 μg/ml T-DM1 with or without metformin at 37 °C for 30 min. After washing, cells were fixed with 3.7% formaldehyde and permeabilized with 0.1% Triton-X 100. The fixed cells were incubated with rabbit anti-caveolin-1 antibody (1:100 dilution in PBS) at room temperature for 1 hour and then incubated with Cy3-conjugated anti-rabbit secondary antibodies (1:200 dilution in PBS) at room temperature for 1 hr. The presence of internalized T-DM1 was revealed by a Cy2-conjugated anti-human secondary antibody. β-Tubulin antibody (Sigma) was used as a marker for T-DM1 efficacy. Coverslips were mounted using Gel Mount aqueous mounting medium (Sigma). Images were acquired using a Zeiss LSM 510 META confocal microscope with a 63× objective (1.4 oil).

### Cell apoptosis analysis

Cell apoptosis was analyzed at 48 hours after drug treatment^1^. Pro-survival protein Bcl-2 was determined by western blot as described previously. Measurement of the extent of apoptosis was carried out using an annexin V detection kit (Invitrogen, ThermoFisher, Waltham, MA, USA) to label phosphatidylserine on the apoptotic cell surface. Briefly, cells were washed once with phosphate-buffered saline, then to each well was added 5 μl annexin V conjugated to fluorescein isothiocyanate (FITC) with binding buffer (10 mM HEPES, pH 7.4, 140 mM NaCl and 2.5 mM CaCl2) in the dark for 15 minutes. Stained cells were analyzed using a Zeiss Axio Observer microscope with a 10× objective. Propidium iodide (PI) was applied to annexin V-stained cells, and cells were observed under a fluorescence microscope to check for necrotic cells.

### NT-DM1 internalization by biotinylation assay

Metformin-pretreated or non-pretreated cells were washed three times with cold PBS, and the membrane was biotinylated using EZ-link Sulfo-NHS-SS-Biotin (Pierce, Rockford, IL, USA) in PBS at 4 °C for 30 min. Labeled cells were washed three times with cold PBS and then incubated with 10 μg/ml T-DM1 or 10 μg/ml T-DM1 with 5 mM metformin at 37 °C for 1 hour for internalization. Biotin remaining on the cell surface was cleaved off by reducing buffer (100 mM sodium-2-mercaptoethane sulfonate, 50 mM Tris, pH 8.6, 100 mM NaCl) at 4 °C, and cells were then scraped into lysis buffer. The biotin-labeled membrane complex with HER2 receptor and T-DM1 was captured on streptavidin ELISA plates (Nunc immobilizer; Nunc, Roskilde, Denmark) from cell lysates diluted to 50 μg/ml total protein in PBS for 30 min. The plates were then washed three times with PBS and incubated with horseradish peroxidase (HRP)-conjugated anti-human secondary antibody for 30 min, and the HRP signal was revealed by incubation with OPD color substrate (Sigma); the reaction was ended by adding 3 M HCl. The level of internalization of T-DM1 was analyzed at 492 nm using an ELISA reader.

### Statistics

The Western blots were quantified by ImageJ software (NIH, Bethesda, MD, USA). Expression level was normalized relative to the quantity of their respective Actin or GAPDH expression, and expressed as multiple of the Control value. Results are expressed as mean ± standard deviation. Student’s t-test was used to compare continuous variables. Differences at the P < 0.05 level were considered statistically significant. The Tukey Kramer test was used for multiple comparisons; in the figures, different letters represent significant differences at the P < 0.01 level. For cell culture experiments, at least three independent experiments were performed.

## Electronic supplementary material

Supplementary Information
