# Supplementary material for: Metformin-induced caveolin-1 expression promotes T-DM1 drug efficacy in breast cancer cells
Source: Sci Rep. 2018 Mar 2;8:3930. doi: 10.1038/s41598-018-22250-8 (PMC5834501; doi:10.1038/s41598-018-22250-8)
Supplement: Supplementary file 1 — Supplementary Information [file 41598_2018_22250_MOESM1_ESM.docx]

**Supplemental Information**

**Metformin-induced caveolin-1 expression promotes T-DM1 drug efficacy in breast cancer cells**

Yuan-Chiang Chung^1#^, Ching-Ming Chang^2#^, Wan-Chen Wei^1,3^, Ting-Wei Chang^3^, King-Jen Chang^4^, Wei-Ting Chao^3*^

^1^ Department of Surgery, Cheng-Ching General Hospital, Chung-kang Branch, Taichung, Taiwan

^2^ Division of Hematology and Oncology, Cheng-Ching General Hospital, Chung-kang Branch, Taichung, Taiwan

^3^ Department of Life Science, Tunghai University, Taichung, Taiwan

^4^ Department of Surgery, Taiwan Adventist Hospital, Taipei, Taiwan

*Corresponding author: Wei-Ting Chao

Email: wtchao@thu.edu.tw

# Equal contribution

Keywords: T-DM1, caveolin-1, metformin, breast cancer


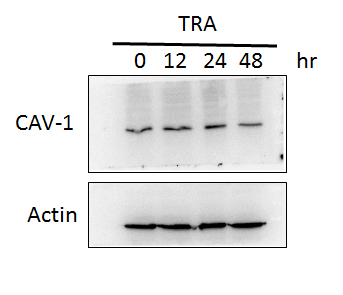


Supplementary Figure S1. Western blot of caveolin-1 expression after trastuzumab treatment. BT-474 cells were treated with trastuzumab (10 μg/ml) for 12, 24 and 48 hours, the cell lysates were collected. The full-length western blots of caveolin-1 and actin from the lysates of the trastuzumab treated cells are shown in the boxed panels (experimental details are described in Methods). The western blots were derived under the same experimental conditions from the same cell lysates.


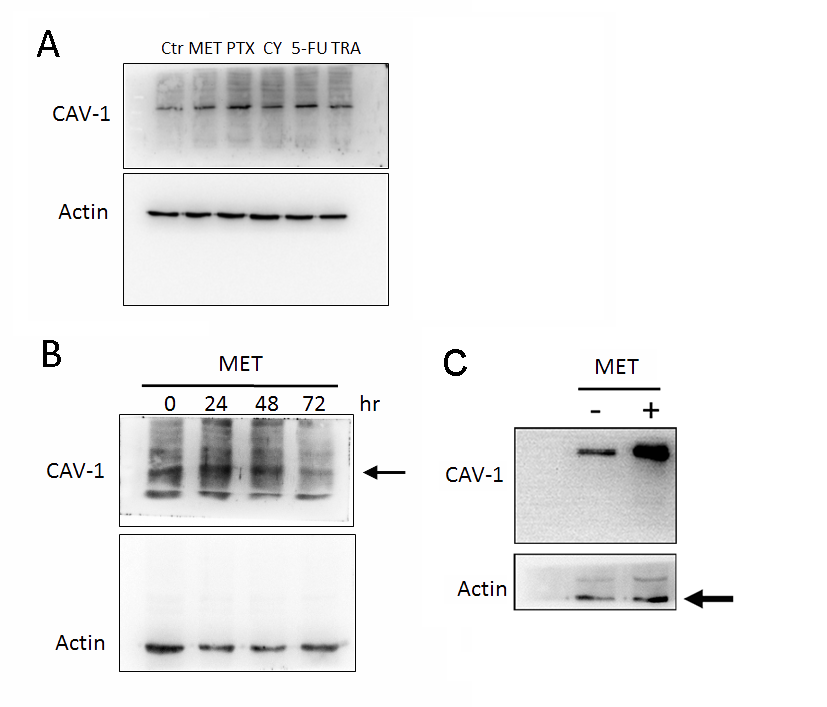


Supplementary Figure S2. (A) Western blot analysis demonstrated caveolin-1 and actin expression when BT-474 cells were treated with metformin, trastuzumab and other chemotherapy drugs for 24 hours. MET: metformin; PTX: paclitaxel; CY: cyclophosphamide; 5-FU: 5-fluorouracil; TRA: trastuzumab. (B) BT-474 cells treated with metformin for 24, 48 and 72 hours. (C) SKBR3 cells treated with metformin for 24 hours. The full-length western blots of caveolin-1 and actin from the lysates of the treated cells are shown in the boxed panels (experimental details are described in Methods). The western blots were derived under the same experimental conditions from the same cell lysates of each treatment group in (A), (B) and (C). Arrows indicate the caveolin-1 and actin shown in Figure 2.


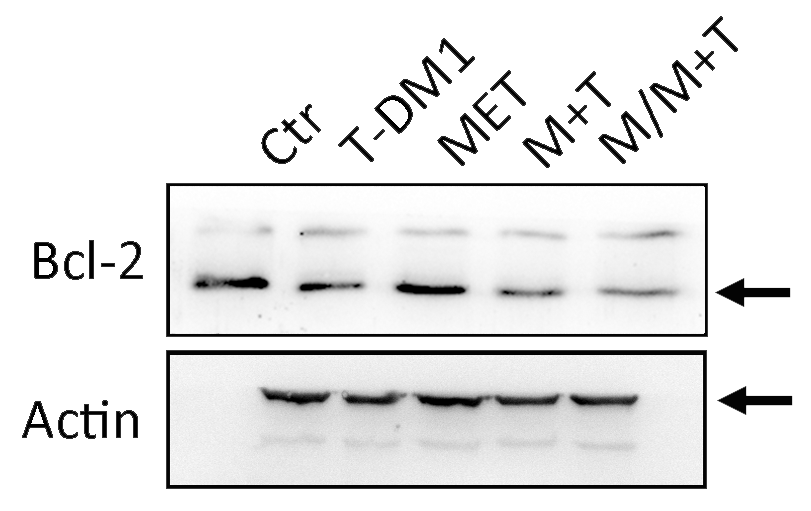


Supplementary Figure S3. Western blot of Bcl-2and actin expression of BT-474 cell pretreated with metformin and then treated with a combination of T-DM1 and metformin. MET/M: metformin; T: T-DM1. Full-length western blots of caveolin-1 and actin from the lysates of the treated cells are shown in the boxed panels (experimental details are described in Methods). The western blots were derived under the same experimental conditions from the same cell lysates. Arrows indicate the Bcl-2 and Actin images shown in Figure 3.


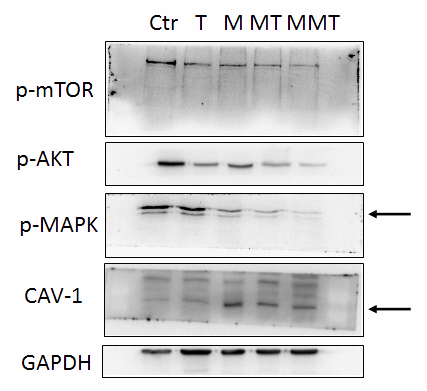


Supplementary Figure S4. Western blots of p-mTOR, p-AKT, p-MAPK, caveolin-1 and GAPDH expression of BT-474 cells pretreated with metformin and then treated with a combination of T-DM1 and metformin. Ctr: control; M: metformin; T: T-DM1. Full-length western blots of p-mTOR, p-AKT, p-MAPK, caveolin-1 and GAPDH from the lysates of the treated cells are shown in the boxed panels (experimental details are described in Methods). The western blots were derived under the same experimental conditions from the same cell lysates. Arrows indicate the p-MAPK and caveolin-1 shown in Figure 5 A.


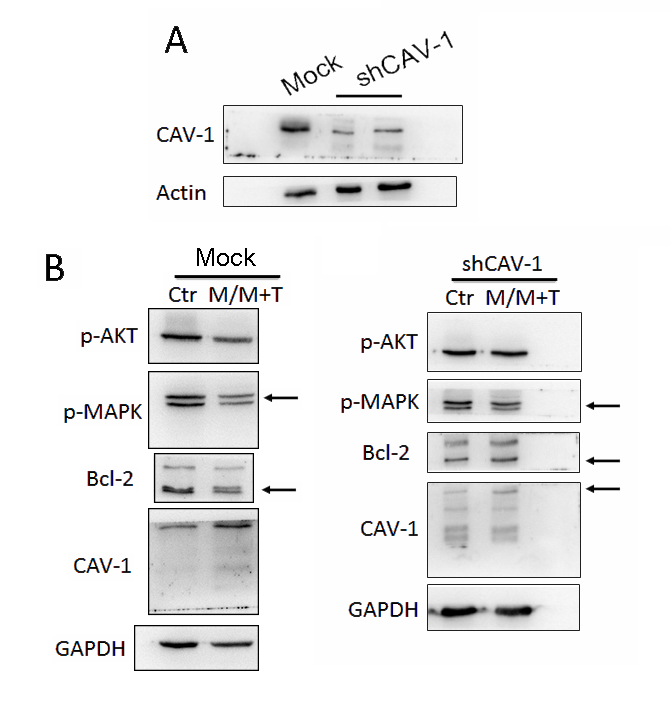


Supplementary Figure S5. (A) Western blot of caveolin-1expression and GAPDH in caveolin-1shRNA treated BT-474 cells. (B) Mock and caveolin-1shRNA treated BT-474 cells were pretreated with metformin and then treated with a combination of T-DM1 and metformin. Ctr: control; M: metformin; T: T-DM1. Full-length western blots of p-AKT, p-MAPK, Bcl-2, caveolin-1 and GAPDH from the lysates of the treated cells are shown in the boxed panels (experimental details are described in Methods). The western blots were derived under the same experimental conditions from the same cell lysates. Arrows indicate the p-MAPK, Bcl-2 and caveolin-1 shown in Figure 5 B and C.


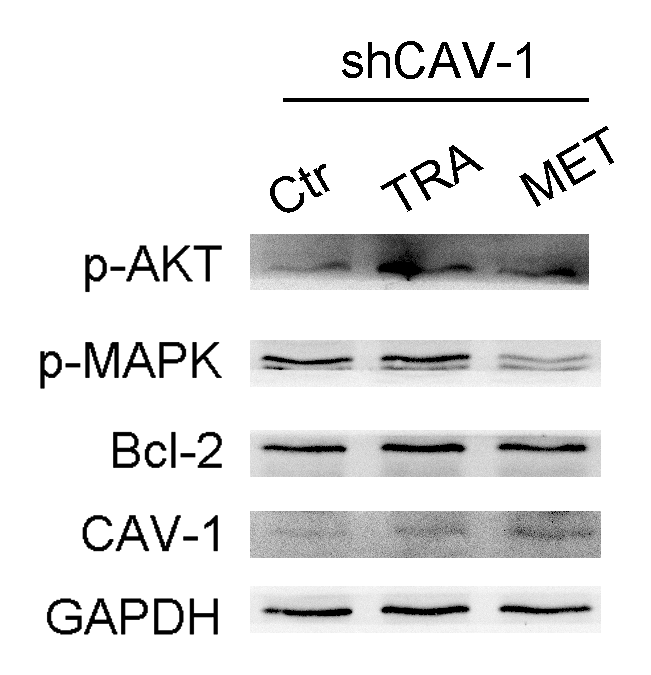


Supplementary Figure S6. The molecular expressions of trastuzumab and metformin treatment in caveolin-1 knocked down cells. BT-474 cells were pre-treated with caveolin-1 shRNA to create caveolin-1-deficient cells. Then, cells were treated with trastuzumab or metformin. The data showed that in caveolin-1 knockdown cells, p-AKT and Bcl2 were not decreased, while p-MAPK was slightly affected. The original full-length western blot images are showed in Supplementary Figure S8. Ctr: control, TRA: trastuzumab, MET: metformin.


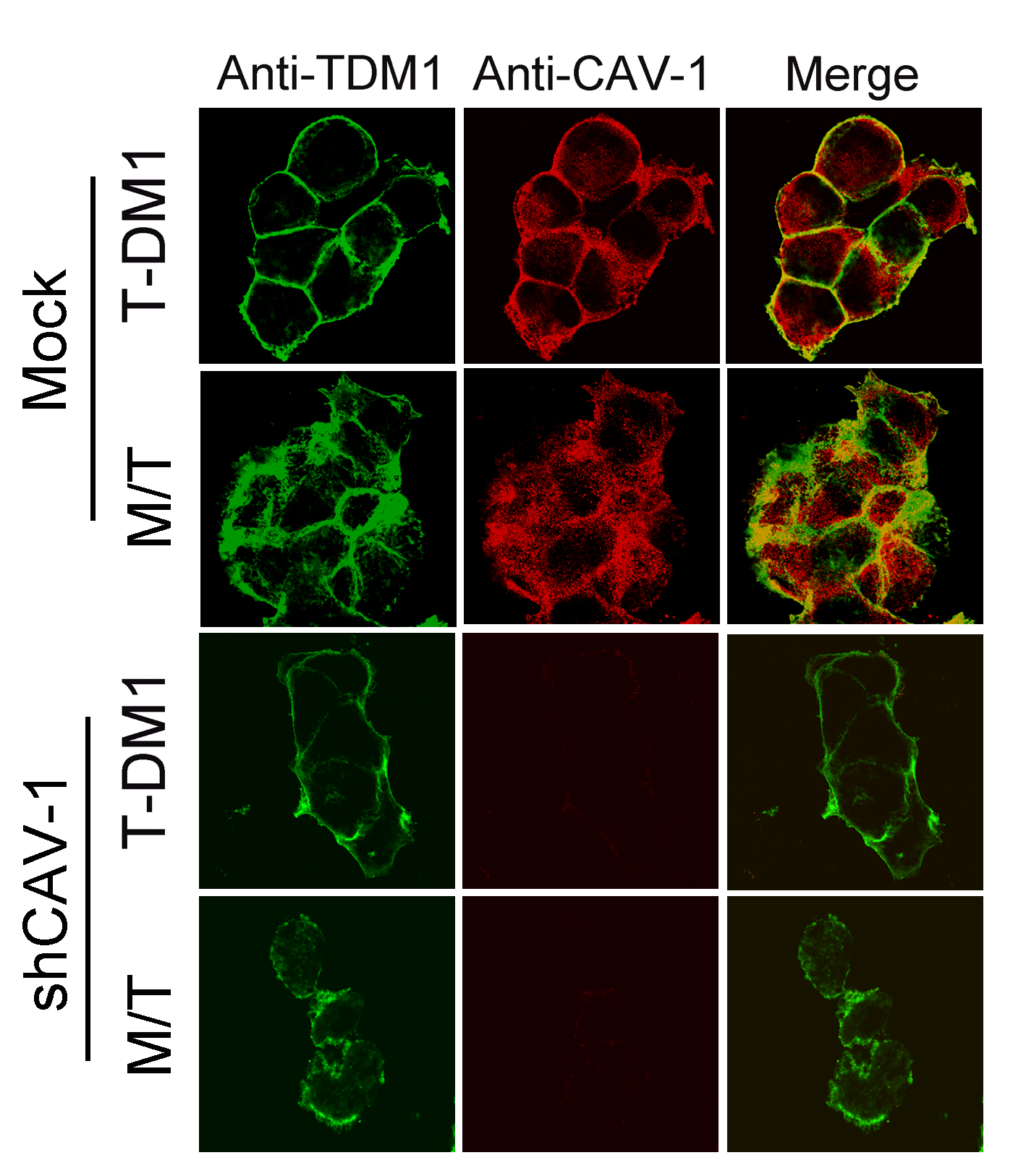


Supplementary Figure S7. Caveolin-1 knockdown inhibited metformin induced T-DM1 internalization. Immuno-confocal microscopy showed that when BT-474 cells were pretreated with metformin and followed by metformin/T-DM1 treatment, caveolin-1 (red) and T-DM1 (green) were co-localized from the cell membrane to the cytoplasm. In caveolin-1 knockdown cells (shCAV-1), after pretreatment with metformin, T-DM1 was distributed on the membrane.


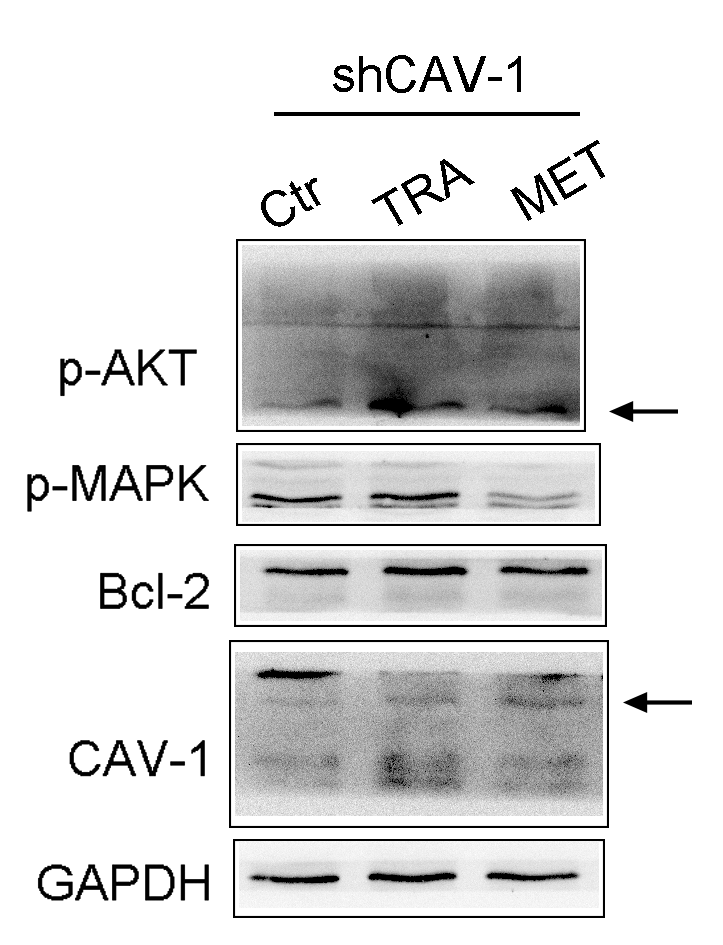


Supplementary Figure S8 Western blot of molecular expressions in caveolin-1 knockdown cells that treated with trastuzumab and metformin. BT-474 cells were treated with caveolin-1 shRNA to create caveolin-1-deficient cells. Then, cells were treated with trastuzumab or metformin. Full-length western blots of p-AKT, p-MAPK, Bcl-2, caveolin-1 and GAPDH from the lysates of the treated cells are shown in the boxed panels (experimental details are described in Methods). The western blots were derived under the same experimental conditions from the same cell lysates. Arrows indicate the p-AKT and caveolin-1 shown in supplementary figure 6.
